# Supplementary material for: Plasma pesticide residues–serum 8-OHdG among farmers/non-farmers diagnosed with lymphoma, leukaemia and breast cancers: A case-control study
Source: PLoS One. 2024 Oct 22;19(10):e0295625. doi: 10.1371/journal.pone.0295625 (PMC11495580; doi:10.1371/journal.pone.0295625)
Supplement: Attachment — Submitted filename: Response to the reviewers.doc [file pone.0295625.s002.doc]

Manuscript title: **Plasma pesticide residues – serum 8-OHdG among farmers/non-farmers diagnosed with lymphoma, leukaemia and breast cancers: a case-control study**

Manuscript ID: **PONE-D-23-18182**

**Authors:** Arun Pandiyan, Summaiya Lari, Janardhan Vanka, Balakrishnan Senthil Kumar, Sudip Ghosh, Babban Jee and **Padmaja R. Jonnalagadda***

At the outset, all the authors wish to thank the Editor, PLOS ONE, and all the esteemed reviewers for their valuable comments and suggestions to improve the quality of the manuscript. Further, the authors have made every effort to address the comments to each point raised by the academic editor and reviewer(s).

**Editor comments:**

| **Comments** | **Response** |
| --- | --- |
| **Comment 1:** Please ensure that your manuscript meets PLOS ONE's style requirements, including those for file naming. | Yes, the revised manuscript meets PLOS ONE's style requirements, including those for file naming. |
| **Comment 2:** Thank you for stating the following financial disclosure:  “This work was supported by Department of Health Research, Ministry of Health & Family Welfare, Government of India under grant R.11012/17/2017-HR”  Please state what role the funders took in the study. If the funders had no role, please state: "The funders had no role in study design, data collection and analysis, decision to publish, or preparation of the manuscript."  If this statement is not correct you must amend it as needed.  Please include this amended Role of Funder statement in your cover letter; we will change the online submission form on your behalf. | Authors wish to mention that the funding agency had no role except that it provided financial grant to undertake the study. Hence the following statement has been included in the cover letter:  "The funders had no role in study design, data collection and analysis, decision to publish, or preparation of the manuscript." |
| **Comment 3:** Thank you for stating the following in the Acknowledgments Section of your manuscript:  “The authors would like to thank the Department of Health Research, Ministry of Health & Family Welfare, GoI, for providing the financial assistance. The authors also acknowledge the support provided by Dr. R. Hemalatha, Director, ICMR-NIN and Dr. N. Jayalatha, Director, MNJ Institute of Oncology & Regional Cancer Institute, Hyderabad and the healthcare officials and paramedical staff, for their helpful co-operation in conducting the study.”  We note that you have provided funding information that is currently declared in your Funding Statement. However, funding information should not appear in the Acknowledgments section or other areas of your manuscript. We will only publish funding information present in the Funding Statement section of the online submission form.  Please remove any funding-related text from the manuscript and let us know how you would like to update your Funding Statement. Currently, your Funding Statement reads as follows:  “This work was supported by Department of Health Research, Ministry of Health & Family Welfare, Government of India under grant R.11012/17/2017-HR” Please include your amended statements within your cover letter; we will change the online submission form on your behalf. | As per the Editors’ suggestion, the funding-related text has been removed from the Acknowledgments section or other areas of the manuscript (Page 25; Line number 434; Section Acknowledgments). However, as per the journal format, this could appear in the funding statement section of the online submission form.  The Funding Statement stands correct. No update is required in the same. |
| **Comment 4:** In your Data Availability statement, you have not specified where the minimal data set underlying the results described in your manuscript can be found. PLOS defines a study's minimal data set as the underlying data used to reach the conclusions drawn in the manuscript and any additional data required to replicate the reported study findings in their entirety. All PLOS journals require that the minimal data set be made fully available. For more information about our data policy, please see http://journals.plos.org/plosone/s/data-availability.  Upon re-submitting your revised manuscript, please upload your study’s minimal underlying data set as either Supporting Information files or to a stable, public repository and include the relevant URLs, DOIs, or accession numbers within your revised cover letter. For a list of acceptable repositories, please see http://journals.plos.org/plosone/s/data-availability#loc-recommended-repositories. Any potentially identifying patient information must be fully anonymized.  Important: If there are ethical or legal restrictions to sharing your data publicly, please explain these restrictions in detail. Please see our guidelines for more information on what we consider unacceptable restrictions to publicly sharing data: http://journals.plos.org/plosone/s/data-availability#loc-unacceptable-data-access-restrictions. Note that it is not acceptable for the authors to be the sole named individuals responsible for ensuring data access.  We will update your Data Availability statement to reflect the information you provide in your cover letter. | The supporting information file is uploaded along with manuscript as a **supplementary file.** However, as per the ICMR guidelines, no patient’s background information can be provided as the study was undertaken after obtaining institutional ethical clearance from both ICMR-NIN and MNJ (a referral State Government) hospital. |
| **Comment 5:** PLOS requires an ORCID iD for the corresponding author in Editorial Manager on papers submitted after December 6th, 2016. Please ensure that you have an ORCID iD and that it is validated in Editorial Manager. To do this, go to ‘Update my Information’ (in the upper left-hand corner of the main menu), and click on the Fetch/Validate link next to the ORCID field. This will take you to the ORCID site and allow you to create a new iD or authenticate a pre-existing iD in Editorial Manager. Please see the following video for instructions on linking an ORCID iD to your Editorial Manager account: https://www.youtube.com/watch?v=_xcclfuvtxQ | As suggested, the ORCID ID of the corresponding author has been updated in the Editorial Manager. |
| **Comment 6:** Please include a separate caption for each figure in your manuscript. | As per the Editors’ suggestion, a separate caption for each figure was added in the revised manuscript (Page 27; Line numbers 448-450; Section Figure Captions). |
| **Comment 7:** Please upload a copy of Figure 3.1, to which you refer in your text on page 11. If the figure is no longer to be included as parts of the submission please remove all reference to it within the text. | We regret the inadvertent error. The comment is now addressed and the figure is removed and all the figure captions are corrected. |

**Response to the specific comments: Reviewer #1**

| **Comments** | **Response** |
| --- | --- |
| **Comment 1:** In page 7, line 27: Please write….The pesticide residues detected were chlorpyrifos, dimethoate….. | As suggested, the corrections were made in the revised manuscript (Page 1; Line numbers 25 & 26). |
| **Comment 2:** In page 11, line 144-149: The extraction and cleanup procedures did not written properly. Please write the extraction procedures clearly. The amount of sample that the authors have taken for extraction and cleanup, and for liquid liquid extraction how much ACN was taken? It was correct that 200 uL ACN taken for liquid liquid extraction? For reconstraction why the authors have used only 200 uL ACN, it was too low? | Yes, it is correct that 200 µl of acetonitrile was taken for liquid-liquid extraction; acetonitrile was added for protein precipitation. The protein precipitation is generally carried out with 1:1 ratio of plasma : organic solvent used. Here, we have also followed a similar methodology. After protein precipitation, the samples were centrifuged to remove the protein pellet and the supernatant extract is completely evaporated to dryness. The residue was reconstituted in 200 µL acetonitrile. As the extraction work was carried out in the microcentrifuge vials, it is possible to reconstitute the residue using 200 µL of acetonitrile, and vortexing followed by centrifugation and also to facilitate the smooth extraction and subsequent transfer of extract which is more than sufficient for LC-MS analysis as we need only 10 µL of sample for injection. The reconstitution value was kept equal to the initial volume of the plasma, so that the additional corrections for concentrations can be avoided. The obtained values directly represent the concentration of pesticide residues present in the plasma samples. As suggested by the reviewer, extraction and cleanup procedures have been properly re-written in the revised manuscript (Pages 5 & 6; Line numbers 146-176). |
| **Comment 3:** In page 13, line 167-175: The procedures for LOQ estimation was not appropriate. According to SANTE and CODEX guidelines, the LOQ should be the lowest fortification level that is achieved the acceptable accuracy and precision. In the study, the authors did not follow it. In this study, the lowest forfication level was 10 ng/mL. Therefore, the LOQ should be 10 ng/mL. If the authors claim that the LOQ was 2 ng/mL, why they did not spike this amount? It should be considered. | Authors agree with the reviewer’s suggestion that according to SANTE and CODEX guidelines, the LOQ should be the lowest fortification level that is achieved the acceptable accuracy and precision. However, in the present study, the LOQ values were calculated correctly in this method. This information was earlier provided in the manuscript in line number 166 and 167 of page 6, where the concentrations used for linear calibration curve were clearly mentioned. The linearity was conducted from 1 to 2000 ng/mL concentration levels. The LOQ values were identified where the precision for 5 numbers of samples falls within 20% deviation levels (as per SANTE, CODEX, FDA and ICH Q2R1 guidelines). Hence in the table, the values were mentioned as 2-10 ng/mL. Therefore, the given values are justified. |
| **Comment 4:** It would better for the readers if the authors provide any justification why they have found higher levels of pesticide residues in the farmers who have leukemia? Is there any relationship among the nature of cancer? | Interestingly, the subjects diagnosed with leukemia had higher levels of pesticide residues. However, a clear-cut relationship with leukemia could not be established possibly these subjects may have slow/low clearance rate of these pesticide residues as compared to other types of cancers. |
| **Comment 5:** In table 1.3: The LOQ was 2-10 ng/mL. However, in table 1.5, the authors have presented positive samples containing residues below the LOQ levels (0.28- 1.38 ng/mL). It was not appropriate. The positive samples should have residues at least LOQ levels or above LOQ levels. The Positive samples are not estimated based on the LOD. Therefore, it is necessary to change it. | The authors would like to clarify the reviewer that in the present study the concentration values in the plasma samples were calculated with regression equation obtained in the linear calibration curve. Hence, the values were obtained below LOQ levels. However, for confirmation, the studies were also conducted using 600 µL of plasma samples and protein precipitation was carried out with 600 µL of acetonitrile and the sample was centrifuged at 10000 rpm for 5 min, the supernatant solution was evaporated to dryness and the residue was reconstituted using 200 µL of acetonitrile. This is 3 times concentration and the values will fall within the linear calibration curve levels. The instrument linearity is 1-2000 ng/mL, however, for confirmation with respect to clinical samples, if the concentration of analytes was not within the detectable range, 600 µL of plasma samples (3x concentration) was used. The same is now incorporated in the revised manuscript (line no. 173 – 176). |
| **Comment 6:** In table 1.3: The linear range was 1-2000 ng/mL, it means that the LCL was 1 ng/mL. How the authors have quantify below that levels (0.28- 1.38 ng/mL; in table 1.5)? | Authors agree with the reviewer’s observation that the LCL for the method was 1 ng/mL. However, we would like to mention that the lowest concentration can be analysed with the same method by increasing the initial volume of the plasma samples. Hence, the presented levels are justified. |

**Response to the specific comments: Reviewer #2**

| **Comments** | **Responses** |
| --- | --- |
| **Comment 1:** Should include 8-OHdG in abstract and intro. Why did the author use 8-OHdG as markers of damage instead of other markers? Lack of explanation about this. | A brief introduction and an explanation on the rationale for studying 8-OHdG in the present study are included in the revised manuscript along with relevant references as suggested (Page 3; Line numbers 71-77). |
| **Comment 2:** Abstract: The pesticide residues in farmers vs non-farmers, significant or not? | Authors wish to mention that in the present study, none of the non-farmers and the healthy controls were detected with any pesticide residues in the plasma samples and hence the reason no statistical comparison could not be performed. The same is reflected in the present manuscript in the section abstract (Page 1; Line numbers 29 & 30). |
| **Comment 3:** Please include ethical approval number/reference. | As suggested, the ethical approval number/reference numbers are included in the revised manuscript (Page 3; Line numbers 85-87). |
| **Comment 4:** Research methodology: What is the percentage/number of non-farmers with similar cancers as the study population i.e. the farmers? | The number of non-farmers with similar cancers as the study population (farmers) were 90 [non-farmers diagnosed with leukemia (n=30), lymphoma (n=30) and breast cancer (n=30)]. The exact number of farmers and non-farmers diagnosed with each cancer are now included in the revised manuscript (Page 4; Line numbers 105-111). |
| **Comment 5:** What are the occupation of non-farmers and control populations? | The occupation of the non-farmers and control population ranged across various sectors such as transport, household services, etc. except getting engaged in any of the farming activities. We would like to emphasize that careful consideration was given while collecting the questionnaire data and samples that none of the subjects who had history of exposure to pesticides were included under the category of non-farmers and control groups. However, the healthy controls are subjects belonging to the family members of farmers but with no exposure to pesticides at any given point of time (Page 4; Line numbers 111-114). |
| **Comment 6:** Should explain what is *pucca*, semi-*pucca* and *kutcha* for the benefit of readers. | Inadvertent error is highly regretted. The parameters for ‘*pucca’*, ‘semi-*pucca’* and ‘*kutcha*’ are explained as foot notes in the Table 1.2 (Page 12; Line numbers 275-276). |
| **Comment 7:** The location away from farm, what is the operational definition? | Since establishing such operational definitions would make the data collection process tedious as it takes time to explain the farmers each question, no such operational definitions were followed in the present study. Hence, the location of the house was considered “In the farm”, if the house was located within the field or at close proximity to the field, the exposure to pesticides would be relatively more as compared to those living “Away from the farm”. |

**Response to the specific comments: Reviewer #3**

| **Comments** | **Responses** |
| --- | --- |
| **Comment 1:** While the chosen topic is captivating, the paper occasionally confuses the study design between cross-sectional and case-control methods. A case-control study should be presented Odds Ratio and 95% confidence interval (95% CI) associated with the exposure covariates by fitting multiple logistic regression models. Logistic regression modeling, in its various forms, has become by far the most frequently applied method for multivariable analysis of case-control studies. Additionally, the inclusion of matching factors during sample recruitment is essential. | We highly appreciate the recommendations. The standard study design of “Hospital based case control study” is followed throughout the revised manuscript. As per the suggestion, we performed both multiple regression and logistic regression and we did not find any significant association between pesticide residue levels or serum 8-OHdG and other confounding variables such as duration of exposure, age, gender, usage of PPE, dietary habits, personal habits such as smoking, drinking alcohol, consumption of tobacco etc. |
| **Comment 2:** In the Methodology section, critical details are missing. To bolster transparency, it’s crucial to include the ethical approval number (line 81). | As suggested, the ethical approval numbers are included in the revised manuscript (Page 3; Line numbers 85-87). |
| **Comment 3:** Further clarification is required regarding how samples were recruited from non-farmers and healthy individuals. Detailed explanations will facilitate a better understanding of the participant selection process. | The authors wish to mention that the detailed explanation for the above comment is clearly explained under the section ‘Inclusion and Exclusion Criteria’ and any further explanation would mean redundancy (Page 4; Line numbers 105-114). |
| **Comment 4:** The basis for the 81% prevalence rate used in the sample size calculation must be referenced to substantiate its validity. The formula chosen must be using expected proportion in both the case group and control group. | A detailed explanation on the estimated prevalence, and determination of the sample size and categorization of the participants is given under the section “Study population and Sampling” (Pages 3 & 4; Line numbers 92-103) of the revised manuscript. We followed the scientifically established statistical methods to determine the sample size with the help of a qualified biostatistician. We further wish to inform that we used the prevalence formula for estimating the sample size i.e.  n = (z)^2^ p (1 – p) / d^2^  From the given formula, we obtained n ≈ 89.33464 and accordingly the sample size was 90. However, since a stratified random sampling technique was adopted with 2:1:1 ratio, the sample size was fixed allocated accordingly at a proportion of 180 (farm workers), 90 (non-farm workers), and 90 (healthy controls), resulting to a total sample size of 360 participants and the same is described in the manuscript. |
| **Comment 5:** A clear description of the process for conducting stratified random sampling is necessary. | The description of sampling is provided in the revised manuscript (Page 4; Line numbers 99-103). |
| **Comment 6:** Figure 2 in line 97 lacks clarity and does not align with the context. The Figure 2 provided was not related. | All the updated figures with relevant captions and texts are now included in the revised manuscript (Page 25-27, Line numbers 438 – 450). |
| **Comment 7:** In Line 104, it is essential to address whether healthy controls living with farmers might also have been exposed to contaminants due to shared living environments. | We would like to emphasize that careful consideration was given while collecting the questionnaire data and the samples from the healthy control groups belonging to the family members of the farmers and also living with them who had neither history of exposure to pesticides nor contaminants. In the present study, we have considered only the occupational exposure as the primary criterion. |
| **Comment 8:** I wonder if you could clarify the status of the validity and reliability of the questionnaire. Particularly in the fifth section, pertaining to knowledge attitude and practice. I also wonder do you need to add the fourth and fifth sections in the methodology, if you are not going to report the result in this paper. The fourth section includes personal information such as dietary habits, consumption of alcohol and smoking were not presented. | We would like to confirm that the questionnaire was pre-tested and validated with around 30 participants prior to initiating the present study. The information related to personal and dietary habits are incorporated in the revised manuscript (Pages 10 & 11; Line numbers 268-273). |
| **Comment 9:** Analysis data for the case-control study were calculated by odds ratio. For multivariate analysis, consider employing Multinomial Logistic regression, to identify which factors influence cancer. | It is a very well-known fact that since cancer is a multifactorial degenerative disorder, without considering several other covariates that influenced the progression of the same throughout the participant’s lifetime, performing regression modelling in this particular study that emphasize only on occupation related factors would be of no consequence. |
| **Comment 10:** Line 222, suggests regression analysis. However, only correlations have been performed. Clarify the discrepancy. | We would like to clarify that the regression analysis was performed for the said variables such as age, gender, personal habits and duration of exposure. However, since there found no significant association between the serum 8-OHdG and the above-mentioned variables, the data were not represented considering the fact that there are nine tables in the manuscript. The phrase (data not represented) is now included in the manuscript (Page 21; Line numbers 327-331). |
| **Comment 11:** Line 238, none of the farmers reported using any PPE. Specify the Personal Protective Equipment (PPE) types for agriculture that you ask your respondents. | The personal protective equipment type is specified in the revised manuscript (Page 10; Line numbers 266-268). |
| **Comment 12:** Table 1.2 Why only demographic factors among cancers? How about non-farmers and healthy subjects? Consider including statistical tests such as Chi-Square or One-way ANOVA to compare between groups. | Since non-farmers and healthy subjects did not share any of the similar parameters related to occupational exposure to pesticides or demographic parameters related to agriculture such as land holding, duration of spraying activity etc., only the data related to farmers were presented. Hence, the statistical tests such as Chi-square or ANOVA were not considered. |
| **Comment 13:** Define terms such as “*Pucca*” and “*Kutcha*” in English terms. Clarify distances denoted by away from the farm. How many kilometers away? What is the meaning of Primary in Educational status? Are they any from secondary? | Inadvertent error is highly regretted. The parameters for ‘*pucca’*, ‘semi-*pucca*’ and ‘*kutcha’* are explained as foot notes in the Table 1.2 (Page 12; Line numbers 275-276). Here, in the present study, the distance from their respective house and the farms can range from 1 km to 5 km. In few instances, some of them stay in the farm (farm houses) was considered in the case of farmers. Hence, the location of the house was considered “In the farm”, if the house was located within the field or at close proximity to the field, since the exposure to pesticides would be relatively more as compared to those living “Away from the farm”. |
| **Comment 14:** Line 260 stated that non-farmers and healthy controls were not detected with residue in Table 1.4, however, there is a wrongly labelling table here. Maybe you want to refer to Table 1.5. Table 1.5 did not have the value for non-farmers and healthy. | Inadvertent error is highly regretted. The caption for the table is now changed to Table 1.5 in the revised manuscript. We would like to mention that none of the non-farmers and healthy controls was detected with any of the pesticide residues in their plasma samples and hence the values were not represented in the table. However, the same was indicated in the revised manuscript (Page 19 & 20). |
| **Comment 15:** Describe the method used to assess data normality. | The Shapiro-wilk test was performed to assess the normality of the data prior to conducting the analysis and the same are incorporated in the revised manuscript under the title “Statistical analysis” (Page 10; line numbers 242-243). |
| **Comment 16:** Explain the rationale behind different exposure cutoff points for various cancer types in Table 1.6. Clearly indicate the unit for pesticide residue levels. | The farmers were categorized into two groups based on the number of years of exposure (i.e., high and low) and the cut off points arrived for leukemia, lymphoma and breast cancers were 14, 20 and 18 years of exposure respectively. The units for pesticide residue levels are now clearly indicated in Table 1.6 of the revised manuscript (Page 20). |
| **Comment 17:** Consider relocating Tables 1.3 and 1.4 to the appendix as their relevance to the discussion is unclear. | The comment is appreciated; however, we would like to clarify that, Table 1.3 and 1.4 are highly vital to the method validation sub-topic and are essential for cross-verifying the parameters discussed under methodology part. |
| **Comment 18:** Under topic Serum 8-hydrxy-2’deoxyguanosine. The numbering of the line has been reduced, For Line 32, there is no statistical analysis conducted, maybe Two-way Anova for farmers and non-farmers and three types of cancer. | The line numbering is properly carried out in the revised manuscript. We would like to clarify that as mentioned in line 241, we carried out the student-T test between three groups and found significant difference (p<0.01) in the levels of 8-OHdG. However, for graphical representation, we have used the violin plot instead of depicting the data in the form of table since the manuscript already has nine tables in total. |
| **Comment 19:** Line 43 to line 47 is only written in text, no table. The reader will be quite confused. Please explain. | Since there found no significant association between the serum 8-OHdG and other confounding variables, the data were not represented considering the fact that there are already nine tables in the manuscript. The phrase (data not represented) is now included in the manuscript (Page 24; Line numbers 327-331). |
| **Comment 20:** Table 1.7 requires a clearer title to indicate the variables being compared. The title should be self-explanatory. Line 56, showed a significant correlation, not a difference. | The title for Table 1.7 is now changed in the revised manuscript (Line number 321) and the said changes were incorporated in Line number 323. |
| **Comment 21:** The authors cite a lot of literature to make the discussion more comprehensive. Authors can streamline the content to make the findings and contributions of the study clearer. | We would like to inform that all the literature citied in the discussion section are essentially vital as supportive studies for the findings of the present study, basically being a comprehensive study on human exposure. Hence, we request the reviewer to consider them as such. However, the study findings and contributions are now rephrased clearly in the revised manuscript (Pages 24 & 25; Line numbers 415-432). |
| **Comment 22:** In Lines 88-90, where is the data or result? Should be presenting it here. | We would like to reiterate that none of the confounding factors showed significant correlation or association with serum 8-OHdG and the data is not presented due to constraints on word count and over representation in the form of tables. |
| **Comment 23:** Provide a concise conclusion that summarizes the key findings and implications of the study. | A concise conclusion summarizing the key findings and implications of the study is addressed and the same is incorporated in the revised manuscript (Pages 24 & 25; Line numbers 415-432). |
